# Factors associated with the use of cognitive aids in operating room crises: a cross-sectional study of US hospitals and ambulatory surgical centers

**DOI:** 10.1186/s13012-018-0739-4

**Published:** 2018-03-26

**Authors:** Shehnaz Alidina, Sara N. Goldhaber-Fiebert, Alexander A. Hannenberg, David L. Hepner, Sara J. Singer, Bridget A. Neville, James R. Sachetta, Stuart R. Lipsitz, William R. Berry

**Affiliations:** 1 Department of Health Policy and Management, Harvard T.H. Chan School of Public Health, 677 Huntington Avenue, Boston, MA 02115 USA; 2 Department of Anesthesiology, Perioperative and Pain Medicine, School of Medicine, Stanford University, Stanford, CA USA; 3 Ariadne Labs at Brigham and Women’s Hospital and the Harvard T.H. Chan School of Public Health, Boston, MA USA; 4 Department of Anesthesiology, Perioperative and Pain Medicine, Brigham and Women’s Hospital, Harvard Medical School, Boston, MA USA

**Keywords:** Operating room crises, Cognitive aids, Crisis checklists, Emergency manuals, Implementation of innovations, Organizational context, Implementation process, Implementation pathway, Quality improvement

## Abstract

**Background:**

Operating room (OR) crises are high-acuity events requiring rapid, coordinated management. Medical judgment and decision-making can be compromised in stressful situations, and clinicians may not experience a crisis for many years. A cognitive aid (e.g., checklist) for the most common types of crises in the OR may improve management during unexpected and rare events. While implementation strategies for innovations such as cognitive aids for routine use are becoming better understood, cognitive aids that are rarely used are not yet well understood. We examined organizational context and implementation process factors influencing the use of cognitive aids for OR crises.

**Methods:**

We conducted a cross-sectional study using a Web-based survey of individuals who had downloaded OR cognitive aids from the websites of Ariadne Labs or Stanford University between January 2013 and January 2016. In this paper, we report on the experience of 368 respondents from US hospitals and ambulatory surgical centers. We analyzed the relationship of more successful implementation (measured as reported regular cognitive aid use during applicable clinical events) with organizational context and with participation in a multi-step implementation process. We used multivariable logistic regression to identify significant predictors of reported, regular OR cognitive aid use during OR crises.

**Results:**

In the multivariable logistic regression, small facility size was associated with a fourfold increase in the odds of a facility reporting more successful implementation (*p* = 0.0092). Completing more implementation steps was also significantly associated with more successful implementation; each implementation step completed was associated with just over 50% higher odds of more successful implementation (*p* ≤ 0.0001). More successful implementation was associated with leadership support (*p* < 0.0001) and dedicated time to train staff (*p* = 0.0189). Less successful implementation was associated with resistance among clinical providers to using cognitive aids (*p* < 0.0001), absence of an implementation champion (*p* = 0.0126), and unsatisfactory content or design of the cognitive aid (*p* = 0.0112).

**Conclusions:**

Successful implementation of cognitive aids in ORs was associated with a supportive organizational context and following a multi-step implementation process. Building strong organizational support and following a well-planned multi-step implementation process will likely increase the use of OR cognitive aids during intraoperative crises, which may improve patient outcomes.

**Electronic supplementary material:**

The online version of this article (10.1186/s13012-018-0739-4) contains supplementary material, which is available to authorized users.

## Background

The operating room (OR) is a high-acuity patient care environment in which crises, although infrequent, occur more commonly than in many other patient care settings. These high-stakes events require rapid, accurate, and coordinated execution of critical steps that can save patient lives. Such crises challenge even the best OR teams since they must optimally handle situations they rarely encounter. Judgment and decision-making can be compromised because of problems with memory retrieval, fixation on an incorrect problem, loss of situational awareness, or other cognitive problems due to stress [1, 2]. OR crisis response can suffer from failure to access knowledge under stress [1, 3] or from poor team communication [1], both of which may result in deviance from established guidelines [4].

Operating room cognitive aids draw from the experience of other high reliability industries such as aviation and nuclear power that use cognitive aids such as checklists and emergency operating procedures to support performance during crises [5–7]. OR cognitive aids serve multiple functions including aiding memory, facilitating decision-making, standardizing action, and standardizing information sharing [8]. The aids do not work in isolation and require a supportive systemic and cultural environment for a successful emergency response in a complex sociotechnical system [8].

Emergency manuals developed by Stanford University School of Medicine (SOM) (Stanford, CA) and OR crisis checklists developed by Ariadne Labs (Boston, MA) are examples of tools designed to support teams during a crisis in the OR. The infrequent occurrence of crises in the OR, cockpit, and nuclear power plant make studying these interventions challenging, and all three industries have turned to simulated environments to generate evidence and refine the use of the cognitive aids. A growing body of evidence suggests the importance of using cognitive aids during OR crises [9–16]. A recent systematic review of simulation studies examining cognitive aid use in diverse OR crises ranging from cardiac arrest to malignant hypothermia found fewer errors and greater efficiency in the management of critical events in 10 out of 13 studies with cognitive aid use [12]. In two of the three studies that did not demonstrate higher performance, clinicians had not been trained on the use of OR cognitive aids [2].

Achieving benefits in patient care through the use of cognitive aids requires that OR teams use them during crises. However, traditional notions of surgery are hostile to memory aids [17], individuals resist changing their routines [18], and long-standing hierarchies undermine the team-based care promoted through OR cognitive aids [19]. Research also suggests that putting innovations such as cognitive aids into practice is challenging [20–22].

Adoption and distribution of cognitive aids in ORs is not enough. For patients experiencing a crisis, the use of the cognitive aids by a well-trained team is what matters. Klein and Knight [23] highlight the distinction between an organization’s decision to adopt an innovation and its implementation, i.e., the consistency and quality of its use. Many innovations are adopted, but not successfully implemented [24, 25]. Implementation research points to some common factors for successful innovation implementation, such as a receptive implementation climate, leadership support, attention to the implementation process, and adequate resources for implementation including time for training [23, 26–29].

While research supports the significant role of implementation strategies in the successful uptake of innovations, little work has been done to explain why some organizations are more successful than others in effectively implementing tools used sporadically like OR cognitive aids for crises.

### Conceptual framework

Our study’s conceptual model draws on prior research and frameworks on health-care innovation implementation [23, 26–29], qualitative research on the WHO Surgical Safety Checklist (SSC) implementation [20–22, 30], and our own experiences with implementing OR cognitive aids in multiple settings [31–33]. We identified two dimensions to be of greatest interest because they are important in the surgical environment and because they are amenable to modification: organizational context and implementation process.

The organizational context dimension refers to the organization’s inner characteristics: how receptive is the organization to the innovation that will be introduced [27]. It includes the facility’s size, teaching status, quality improvement culture, and facilitators and barriers to implementing the cognitive aid [34–40].

An active change process is critical for a successful implementation [27]. We have identified eight key steps to effective implementation of OR cognitive aids including presenting the cognitive aid at meetings, forming a multidisciplinary team, customizing the cognitive aid to the facility’s local context, pilot testing it, providing training, delivering ongoing training, monitoring use of the cognitive aid, and expanding it to areas outside the OR where anesthesia is administered [32].

We designed the study to take advantage of access to potential implementers who have downloaded the tools from the websites of Stanford SOM and Ariadne Labs. We analyzed survey data from respondents representing United States (US) hospitals and ambulatory surgery centers (ASCs) that had initiated the implementation of OR cognitive aids for crises to understand how organizational context and implementation processes influenced implementation effectiveness. For the purposes of this study, we defined implementation effectiveness as reporting consistent use of cognitive aids during OR crises. Our hypothesis was that a supportive organizational context and following a multi-step implementation process would be associated with more successful implementation of OR cognitive aids.

## Methods

### Study design and sample selection

We performed a cross-sectional analysis using data gathered through a Web-based survey to examine factors that might be related to success in implementing OR cognitive aids. Our sampling frame included individuals who had downloaded OR crisis checklists or emergency manuals (tool) from the websites of Ariadne Labs or Stanford SOM (*n* = 12,722) between January 2013 and January 2016. All downloaders were invited to complete a survey described below. Surveys were completed between January and February 2016. A total of 1796 survey responses were collected [response rate = 14.1%]. After removing 96 incomplete responses, 1700 remained for analysis. We focused our analysis on a subset representing facilities whose implementation processes for the tool had existed long enough (6 months or more) to potentially achieve implementation success. Using the survey question, “What is the stage of use of the tool in your facility?”, we removed all respondents who reported that they had only “downloaded the tool, but it is not in use in our operating rooms” (*n* = 709) and those who reported that they were “in the process of deploying the tool to our operating rooms” (*n* = 225). We also excluded those who responded that they “neither agreed nor disagreed” (*n* = 248), indicating possible uncertainty about tool use, and those who did not respond (*n* = 43) to whether the tool is used regularly during applicable clinical events; this survey question was used as the outcome for the study. Finally, we excluded respondents from international hospitals (*n* = 107), as the intent of the work was to understand implementation in the domestic setting, and cultural context can influence implementation [41]. Our final analytic dataset was comprised of 368 surveys from respondents at US facilities.

### Survey development

A five-member multidisciplinary team with diverse backgrounds in anesthesia, surgery, patient safety, health systems innovations, and implementation science developed the survey, with further input from steering committee members of the Emergency Manuals Implementation Collaborative [42].

The 22 survey questions addressed four topics: (1) organizational context factors (five items) including the number of ORs, whether the organization had surgical or anesthesiology residents, the surgery department’s participation in various quality improvement (QI) initiatives, and the facilitators and barriers to implementing the tool; (2) factors related to the implementation process (eight items), such as forming a multidisciplinary team, customizing the tool to the facility’s local context, or providing training; (3) use and motivation factors (four items), including whether the tool was used regularly during OR crises, the stage of implementing the tool, the perceived impact of the tool, and whether the respondent would want the tool used if they were themselves having an intraoperative emergency; and (4) respondent characteristics (three items), including primary role at the facility, years since professional training, and type of employment, as well as facility characteristics (two items) including whether the facility was a hospital or ASC and whether it was located in the USA (see Additional file 1 for the survey).

To ensure that the survey was both comprehensive and understandable, we cognitively tested it with 21 providers whose roles were similar to those of our intended respondents by asking them to read and react to survey items. We revised questions found to be unclear and added questions covering missing topics. We invited individuals in our sample via email and sent up to two reminders at 1-week intervals. We offered to share the survey results with respondents but offered no financial incentives for completing the survey.

### Definition of main outcome

We measured our main outcome, implementation success, using one survey item: “At my facility, the tool is used regularly during applicable clinical events.” We chose this language to allow participants to consider their own threshold for appropriate use. Five response options ranged from “strongly disagree” to “strongly agree” with a neutral midpoint. Respondents who agreed or strongly agreed with the item, meaning they reported regular use of the tool during applicable clinical events, were considered to have “more successful implementation.” Respondents considered to have “less successful implementation” were those who indicated that they disagreed or strongly disagreed with the item. Those who “neither agreed nor disagreed” and those who did not answer were not included in the analysis.

### Creation of covariate composite scores

Our covariates consisted of three summary scores. Each score represented the respondent’s assessment of their facility’s QI experience, implementation processes, and the extent the tool was used for diverse purposes. The QI experience score included prior experience with (a) WHO Safe Surgery Checklist, (b) simulation training, (c) communication and teamwork training, (d) protocols for handoffs, and (e) emergency drills. The implementation process score included having (a) formed a multidisciplinary team, (b) customized the tool, (c) pilot tested the tool, (d) presented the tool at meetings, (e) provided training on the effective use of the tool, (f) provided retraining, (g) monitored the use of the tool, and (h) expanded the use of the tool beyond ORs where anesthesia was administered. The use score reflected that a facility used the tool (a) for emergency drills, (b) to prepare for a complex case, (c) to debrief after a critical event, and (d) for educational review. These three composite scores ranged in value from zero to the number of items in each grouping.

### Analysis

All analyses were performed in SAS, version 9.4 (Carey, NC). All variables and responses were categorical or ordinal and thus described with frequencies and percentages. The relationships between the main outcome (reporting more successful vs. less successful implementation) and covariates were first assessed in bivariate analyses. In these bivariate analyses, Pearson chi-square tests were used for non-ordinal survey items, and Mantel-Haenszel chi-square tests for trend were used for ordinal survey items such as number of ORs and the composite scores. These bivariate analyses were used to identify a set of variables for the multivariable model. We performed multivariable logistic regression to identify significant predictors of the main outcome: reporting more successful implementation. We were especially interested in the relationship between implementation success and facility size [number of ORs], number of implementation steps, and the number of diverse ways in which the tool was used, so we included those three variables in the multivariable model for theoretical reasons. All other potential covariates were assessed and dropped from the multivariable model if they had a *p* > 0.05. Results are presented as odds ratios with 95% confidence intervals (CI) and *p* values.

The relationships between secondary outcomes and the main outcome were assessed in bivariate analyses like those described above. Two-sided *p* values less than 0.05 were considered statistically significant.

## Results

### Facility and respondent characteristics

Table 1 describes the characteristics of 368 respondents and the facilities they represent. In bivariate analyses, we compared respondent characteristics from facilities that reported more successful implementations (*n* = 241, 65.5%) vs. less successful implementations (*n* = 127, 35.5%), respectively. Most of the survey respondents included in our final analysis were anesthesiology providers (almost 85%), worked for a facility/university or physician-owned practice (57.3%), and had been practicing for 20 years or more since their professional training (53.8%). There were no significant differences in these characteristics between respondents reporting more successful vs. less successful implementation.

**Table 1:** Sample characteristics

	All respondents		Less successful implementation		More successful implementation		
	*N* = 368		*N* = 127 (34.5%)		*N* = 241 (65.5%)		
Variables	*n*	%	*n*	%	*n*	%	*p* value
Facility characteristics							
Number of ORs							0.0014*^‡^
0–4	90	24.5	17	13.4	73	30.3	
5–15	119	32.3	43	33.9	76	31.5	
16–30	83	22.6	37	29.1	46	19.1	
≥ 30	74	20.1	30	23.6	44	18.3	
Missing	2	0.5	0	0.0	2	0.8	
Anesthesiology or surgical residents	175	47.6	66	52.0	109	45.2	0.2184
Respondent characteristics							
Primary professional role							0.179
Anesthesiology	311	84.5	114	89.8	197	81.7	
Surgery	13	3.5	2	1.6	11	4.6	
OR staff	24	6.5	7	5.5	17	7.1	
Other	20	0.1	4	0.0	16	0.1	
Employer^†^							0.9687
Facility or university	118	32.1	44	34.7	74	30.7	
Physician owned practice	93	25.3	34	26.8	59	24.5	
Corporate practice	51	13.9	20	15.8	31	12.9	
Independent solo practice	27	7.3	9	7.1	18	7.5	
Missing	79	21.5	20	15.8	59	24.5	
Years since professional training							0.4534^‡^
Less than 5 years	31	8.4	12	9.5	19	7.9	
5–10 years	40	10.9	15	11.8	25	10.4	
11–20 years	80	21.7	29	22.8	51	21.2	
≥ 20 years	198	53.8	66	52.0	132	54.8	
Missing	19	5.2	5	3.9	14	5.8	

*Significant at alpha = 0.05

†Fisher’s exact test

‡Mantel-Haenszel (MH) chi-square test

### Organizational context

#### Structural characteristics

Unadjusted analyses in Table 1 show that teaching status (presence of anesthesiology or surgical residents) (*p* = 0.2184) and facility type (hospital or ambulatory surgical center) (*p* = 0.9687) had no relation to implementation success. There was a significant relationship, however, with the facility size [number of ORs]; respondents that reported more successful implementation had fewer ORs (*p* = 0.0014). The row percentages reveal this trend more intuitively; in facilities with 0–4 ORs, 81.1% (73/90) of respondents reported more successful implementation, compared to 59.5% (44/74) respondents in facilities with at least 30 ORs.

#### Quality improvement initiatives

Respondents that reported more successful implementation were significantly more likely to have QI initiatives in place related to communication and teamwork training (*p* = 0.0200) and emergency drills (*p* < 0.0001) (Additional file 2).

#### Barriers and facilitators

**Fig. 1:**
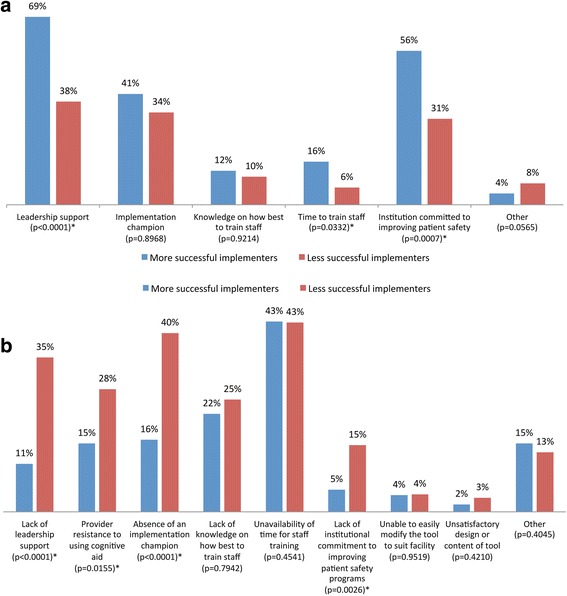
OR cognitive aid implementation facilitators and barriers in facilities with more vs. less successful implementation. **a** Facilitators. **b** Barriers

A higher percentage of respondents from facilities with more successful implementation reported factors enabling implementation including institutional commitment to improving patient safety (*p* = 0.0007), leadership support (*p* < 0.0001), and time to train staff (*p* = 0.0332). Conversely, a higher percentage of respondents from facilities with less successful implementation mentioned barriers to implementing the tool including a lack of institutional commitment to improving patient safety (*p* = 0.0026), lack of leadership support (*p* < 0.0001), absence of an implementation champion (*p* < 0.0001), and provider resistance to using cognitive aids (*p* = 0.0155) (Fig. 1).

### Implementation process

There were significant differences between respondents reporting more successful implementation and those reporting less successful implementation regarding their engagement in each of the eight steps we hypothesized were helpful for implementing the tool (Additional file 2). The following were associated with more successful implementations: the tool was presented at staff, physician, or departmental meetings (*p* < 0.0001); a multidisciplinary team was established to review the tool (*p* < 0.0001); the tool was customized to fit the local context (*p* = 0.0002); the tool was pilot-tested (*p* < 0.0001); providers were trained in the use of the tool (*p* < 0.0001); ongoing/routine training was provided on the tool (*p* < 0.0001); the use of the tool was monitored (*p* < 0.0001); and the use of the tool was expanded to additional areas in the facility outside the OR where anesthesia was administered (*p* < 0.0001).

### Covariate composite scores

Table 2 shows the bivariate associations between composite scores for QI initiatives, implementation steps, and tool use, which all showed significant positive trends with implementation success. Over 70% of respondents whose facilities accomplished all five QI initiatives reported more successful implementation compared to 54.7% with only one QI initiative (*p* = 0.0049). A similar trend was seen for number of implementation steps: 92.3% of respondents whose facilities completed all eight implementation steps reported more successful implementation vs. 25.6% with only one implementation step (*p* < 0.0001). Finally, 91.7% of those who used the tool for all five purposes reported more successful implementation compared to 53.4% using the tool for just one purpose (*p* < 0.0001).

**Table 2:** Unadjusted association between composite scores and facility implementation status (more vs. less successful implementation)

		More successful implementation		
Variable	Total *N*	*N*	%	MH *p* value
Number of patient safety/quality improvement initiatives^†^				0.0049*
1	53	29	54.7	
2	59	32	54.2	
3	89	59	66.3	
4	79	60	76.0	
5	78	55	70.5	
Missing	10	6	60.0	
Number of cognitive aid implementation steps completed^‡^				< 0.0001*
0	39	10	25.6	
1	52	19	36.5	
2	67	41	61.2	
3	60	42	70.0	
4	45	38	84.4	
5	35	27	77.1	
6	42	39	92.9	
7	15	13	86.7	
8	13	12	92.3	
Missing	0	0	0.0	
Number of other ways in which tool is used^§^				< 0.0001*
0	73	39	53.4	
1	146	87	59.6	
2	91	68	74.7	
3	42	36	85.7	
4	12	11	91.7	
Missing	4	0	0.0	

*Significant at alpha = 0.05

^†^The QI initiatives score was calculated by summing the following yes/no questions: (1) WHO Safe Surgery Checklist, (2) simulation training, (3) communication and teamwork training, (4) protocols for handoffs, and (5) emergency drills

^‡^The implementation step score was calculated by summing the following yes/no questions: (1) Has the tool been presented at staff, physician, or departmental meetings, (2) Has your facility established a multidisciplinary team to review the tool, (3) Did you customize the tool to your facility’s local context, (4) Did your facility pilot test the tool, (5) Has your facility trained people working in the use of the tool, (6) Does your facility provide ongoing/routine training on the effective use of this tool, (7) Does your facility monitor the use of the tool, and (8) Has your facility expanded the use of the tool to other areas in the hospital where anesthesia is being administered

^§^The number of ways in which tool is used score was calculated by summing the following yes/no questions: (1) emergency drills in the OR or simulation center, (2) to prepare for a complex case, (3) to debrief after a critical event, and (4) educational review

### Predictors of regular OR cognitive aid use

Table 3 presents the results for the final adjusted multivariable model (*c*-statistic = 0.849). Four organizational factors were associated with reporting more successful implementation: fewer ORs (*p* = 0.0203), more ways in which the tool was used (*p* = 0.0328), leadership support (*p* < 0.0001), and time to train staff (*p* = 0.0189). Small facility size (0–4 ORs compared to referent group of 30 plus ORs) was associated with a fourfold increase in the odds of reporting more successful implementation (odds ratio (OR) = 4.11, 95% confidence interval (CI) = 1.66–10.18, *p* = 0.0092), while leadership support (OR = 3.26, CI = 1.80–5.91, *p* < 0.0001) and dedicated time to train staff (OR = 3.75, CI = 1.24–11.28, *p* = 0.0189) were each associated with at least a threefold increase in the odds of reporting more successful implementation. Three barriers were significantly associated with less successful implementation: resistance to use from clinical providers (OR = 0.18, CI = 0.08–0.38, *p* < 0.0001), absence of a committed implementation champion (OR = 0.44, CI = 0.23–0.84, *p* = 0.0126), and unsatisfactory content or design of the tool (OR = 0.11, CI = 0.02–0.61, *p* = 0.0112). Completing a higher number of implementation steps was significantly associated with more successful implementation. Each implementation step completed was associated with just over 50% higher odds of more successful implementation (OR = 1.57, CI = 1.31–1.87, *p* = < 0.0001).

**Table 3:** Multivariable analysis to identify predictors of regular use of cognitive aids during appropriate OR crises (successful implementation reported)

Predictor	Odds ratio	95% confidence interval	*p* value
Number of implementation steps (out of 8)^†^	1.57	1.31–1.87	< 0.0001*
Number of operating rooms			0.0203*
0–4	4.11	1.66–10.18	0.0092*
5–15	2.20	1.01–4.80	0.6446
16–30	1.69	0.73–3.91	0.5367
≥ 30	Reference group	n/a	n/a
Number of other ways in which tool was used (out of 4)^‡^	1.41	1.03–1.92	0.0328*
Enabled tool implementation—support of my department or institution leadership	3.26	1.80–5.91	< 0.0001*
Enabled tool implementation—time to train staff	3.75	1.24–11.28	0.0189*
Challenge to implement—clinical providers resisted using tool	0.18	0.08–0.38	< 0.0001*
Challenge to implement—absence of committed implementation champion	0.44	0.23–0.84	0.0126*
Challenge to implement—found content or design of tool unsatisfactory	0.11	0.02–0.61	0.0112*

*c*-statistic = 0.849

*Significant at alpha = 0.05

^†^The implementation step score was calculated by summing the following yes/no questions: (1) Has the tool been presented at staff, physician, or departmental meetings, (2) Has your facility established a multidisciplinary team to review the tool, (3) Did you customize the tool to your facility’s local context, (4) Did your facility pilot test the tool, (5) Has your facility trained people working in the use of the tool, (6) Does your facility provide ongoing/routine training on the effective use of this tool, (7) Does your facility monitor the use of the tool, and (8) Has your facility expanded the use of the tool to other areas in the hospital where anesthesia is being administered

^‡^The number of ways in which tool is used score was calculated by summing the following yes/no questions: (1) emergency drills in the OR or simulation center, (2) to prepare for a complex case, (3) to debrief after a critical event, and (4) educational review

There was a significant difference between respondents that reported more successful implementation and those that reported less successful implementation in the ways the tool was used (see Additional file 2). More successful implementation was associated with the use of the tool in emergency drills (*p* < 0.0001), in preparation for complex cases (*p* = 0.0328), and in debriefing after a critical event (*p* < 0.0001).

### Correlation of secondary outcomes with primary outcome

#### Perceptions about the impact of bringing the cognitive aid into the OR

There was a significant difference in the perceptions of the impact of introducing the OR cognitive aid in the operating room in respondents from facilities reporting more vs. less successful implementations. More successful implementation was more frequently associated with the perception that implementing the tools had improved communication during a crisis (*p* < 0.0001), improved teamwork during a crisis (*p* < 0.0001), brought disciplines together to train as teams (*p* < 0.0001), created a system to enable debriefing after crises occur (*p* = 0.0009), helped to identify equipment issues to deal with particular crises (*p* = 0.0056), improved team performance during a crisis (*p* < 0.0001), and improved outcomes from critical events (*p* < 0.0001). On the other hand, less successful implementation was more frequently associated with the perception that there had been no impact from introducing the tool in the operating room (*p* < 0.0001) (Additional file 3).

#### If respondents would like a cognitive aid used if they were a patient

**Fig. 2:**
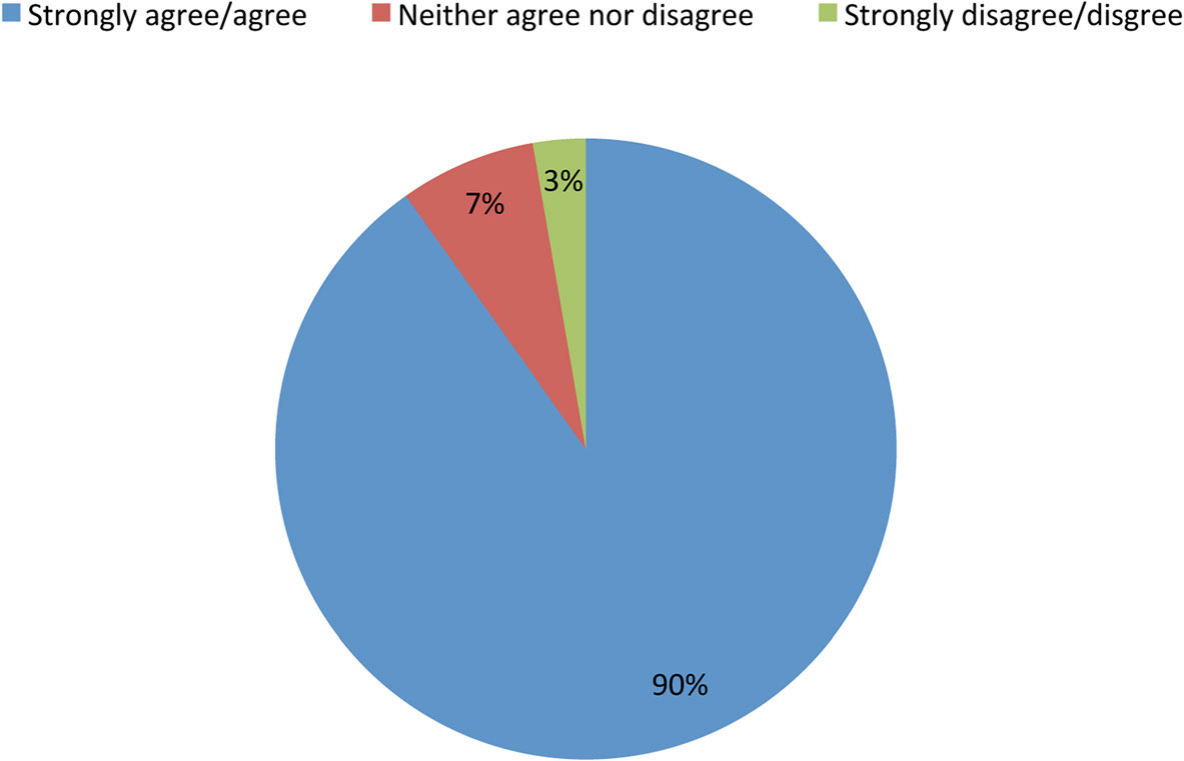
If I had an operation with an intraoperative emergency, I would want this tool used

A total of 90% of respondents, irrespective of their facility’s reported implementation status, agreed or strongly agreed with the statement “If I were having an operation that had an intraoperative emergency, I would want this tool to be used” (Fig. 2).

### Sustainability of implementation

Just under 10% of respondents indicating that they had implemented the tools in their operating rooms agreed with the statement “clinicians used it initially, but we have not been successful in sustaining its use.” We did not ask other specific questions about sustainability.

## Discussion

OR cognitive aids draw from the long experience and accumulated evidence from other high reliability industries such as aviation and nuclear power. They are an important component of an integrated approach to improving care in a complex socio-technical environment, the operating room. While in aviation and nuclear power, these tools have evolved from paper-based to computerized systems, using the expertise of human factors engineers and others to optimize their design and use, in healthcare, they remain as paper-based tools. Despite their early stage of development, OR cognitive aids have demonstrated efficacy in improving management and care in an OR crisis [9–16]. Widespread implementation of these aids in health-care facilities has not occurred, and a better understanding of factors related to the organizational context and the implementation process on implementation success is necessary.

To test our hypothesis that a more supportive organizational context and following a multi-step implementation process would be associated with more successful implementation of OR cognitive aids, we studied the responses of representatives of hundreds of health-care facilities using as our marker of successful implementation and primary outcome variable the respondent’s perception of whether the tool is used regularly during applicable clinical OR events at their facility. We found that respondents from facilities with more successful cognitive aid implementation identified more supportive organizational contexts, including greater participation in quality improvement initiatives, leadership support for implementation, dedicated time for staff training, and being smaller facilities. We also found that respondents from facilities with more successful implementation reported following a greater number of the checklist implementation steps that have been shown to be important in the implementation of other innovations. In contrast, respondents from facilities with less successful implementation reported less supportive contexts, including lack of leadership support, lack of an implementation champion, and provider resistance to using OR cognitive aids, and were less likely to follow all the implementation steps. These findings are consistent with those of retrospective reviews of nearly 500 empirical and non-empirical studies on innovation implementation, which found organizational context and implementation process to be important considerations in successful innovation implementation [26, 27].

Our analyses suggest a set of recommendations that may improve the likelihood of successful implementation of OR cognitive aids, which could improve outcomes for patients who experience crises during surgical procedures.

### Dealing with facility size

Given mixed findings about size [34–37], scholars suggest that the relationship between facility size and innovation might vary based on the type of innovation studied [35, 38]. Our analysis indicates that the implementation of OR cognitive aids was more often achieved in smaller facilities. An innovation such as an OR cognitive aid, which requires a high-touch implementation approach, may be easier to incorporate into the existing processes of smaller facilities. For example, buy-in might be easier to achieve because of the strength of relationships, greater face-to-face communication, and fewer people being needed to support the change. When compared to larger facilities, smaller facilities also tend to be more flexible and less bureaucratic and exhibit less inertia [38]. Larger facilities may have more formalized structures and processes; more hierarchy and greater bureaucracy, including many more rules and procedures; and longer decision chains that could hinder innovation in comparison to smaller facilities [39]. Our experience from implementing the Safe Surgery Checklist suggests that in smaller facilities, a single individual acting as a champion is more likely to drive towards successful implementation. At the same time, in a smaller facility, a single person can successfully block the innovation and prevent its implementation. Since our study focuses on those who downloaded the tool and completed our survey, the favorable relationship between small facility size and implementation may be a result of the presence of an implementation champion, i.e., the downloader. Although the size of a facility cannot be changed, our findings suggest that the implementation of OR cognitive aids in larger facilities might benefit from modeling some of the implementation processes of smaller facilities. Dividing a large facility into smaller units and doing a staged, sequential rollout to progressively gain the acceptance of staff and build consensus behind the innovation’s use might be more effective than a simultaneous full-facility rollout.

### Performing prior quality improvement initiatives

We found that previous quality improvement experience was associated with success in OR cognitive aid implementation: the greater its number of quality improvement initiatives, the more likely a facility was to successfully implement OR cognitive aids. Weiner et al. [40] suggest that greater participation in quality improvement initiatives may create a “quality culture” with shared values about the importance of improvement, the use of systems to prevent error, and the need for communication and coordination to solve problems. This may result in the development of organizational infrastructure and routines, such as the use of teams, extensive training, and an emphasis on data for monitoring [40, 43], which appears to be associated with successful implementation of OR cognitive aids.

Our findings suggest the importance of first understanding a facility’s broader experience with quality improvement when implementing OR cognitive aids to anticipate the greater effort that may be needed when prior experience is lacking. An additional benefit of OR cognitive aid implementation may be that a facility can gain more experience in undertaking quality improvement initiatives and create a quality culture and structures that enable future initiatives.

### Building leadership support

In our analysis, we identified supportive leadership as being an important predictor of successful implementation of OR cognitive aids. The role of senior management in the successful implementation of quality improvement innovations in healthcare and other organizations has long been recognized [43]. Leadership support when implementing OR cognitive aids signals that the innovation is a priority. Engaged leadership is also better aware of what is needed to successfully implement innovations, can ensure a high-quality package of implementation policies and practices, and can help in addressing physician resistance [23, 43]. In addition, informal leadership support provided by respected clinical role models who can engage their peers, communicate the importance of the OR cognitive aids, cultivate buy-in, and demonstrate innovations and practices changes is also critical [44, 45].

### Making time available for training

Not surprisingly, we found that facilities that provided time for training in the use of OR cognitive aids had greater success with implementation than those that provided little or no training. Training is essential for clinicians to gain familiarity and to use the aids effectively. Research shows that clinicians and teams are more likely to utilize OR cognitive aids when they are trained in their use [46–48]. Without sufficient training, providers may put the cognitive aids aside or even misuse them by using the wrong checklist or algorithm [2].

### Enhancing provider willingness to use OR cognitive aids

We found that perceived provider resistance or reluctance to use the aids was associated with less successful implementation of OR cognitive aids when compared to those that supported their use. The perception that content or design of the tool was unsatisfactory was similarly associated with less successful implementation when compared to those that were happy with the content and design. We followed up on our survey results with qualitative interviews with a smaller group of representative survey respondents (*n* = 37). Interview respondents described two key reasons for resistance they encountered when implementing the tools: a belief that consulting a cognitive aid during a crisis would show “weakness” or make the clinicians look incompetent (*n* = 6) and a belief that using the tools is not necessary because the clinicians already know what to do and have everything memorized (*n* = 7). Older anesthesiologists were felt to hold these beliefs more commonly than younger anesthesiologists (*n* = 8). Even among those interviewed respondents who indicated that they did not need the tool themselves, the reason given was not that the tool was inadequate or unproven, but rather that they were so competent that it made use of a tool unnecessary [personal correspondence with W. Berry, December 1, 2017].

In other safety-critical industries, such as aviation and nuclear power, there is cultural acceptance of the use of cognitive aids, training with them is universal, and their use is even mandated in crisis situations. In healthcare, Harrison et al. [11] highlighted barriers that must be addressed to promote consistent cognitive aid use. These barriers include a limited understanding of the vulnerability of the human decision-making process in stressful situations, widely held concerns that providers will be perceived as not knowing what they are doing if they use a cognitive aid, perceptions that their use might be a burden during a time-critical event, and lack of proper design to make tools easily usable. Thus, facility leaders who want to successfully implement OR cognitive aids must build clinician buy-in. Two approaches to creating buy-in include sharing these tools’ helpfulness and immersive training in their use. For example, in a study by Low et al. [49], physicians indicated that training increased their confidence in dealing with a potential clinical event, and after the training, they did not feel that using cognitive aids reflected a lack of competence. The fear that cognitive aid use implies incompetence potentially threatens implementation success. The fear should be addressed during the implementation process.

### Following a multi-step implementation process

Our analysis showed that a multi-step implementation process is important; respondents from facilities with successful implementation noted more steps being performed than those that were less successful, and respondents from facilities with the highest levels of success reported that all the process steps were performed. Implementing cognitive aids requires more than printing a checklist and putting it in the OR, sometimes referred to as “print and plunk.” Our analysis identified that the entire implementation process is associated with effectively rolling out OR cognitive aids, consistent with our previous work that identified key procedural steps as essential for success [32].

One take-away message emerging from our analysis is that it may be beneficial to consider every implementation step before skipping one. They are all important to address in some way, without needing to be long, formal processes. First, it is important to build a receptive implementation climate by recruiting a team, equipping them with checklist information, and assessing local culture and organizational readiness for implementation [50]. Second, adapting the cognitive aid to fit the local context is also critical. Previous studies have described innovations as having a “core” and an “adaptable periphery” [27]. Due to differences in surgical specialties, equipment, drugs, and protocols across hospitals, it is essential to safe care that a facility adapt the OR cognitive aids to fit the local context. Similarly, piloting through dry runs helps ensure that the aid fits the local context, while allowing additional individuals to gain familiarity, increase their confidence, and champion the change [5]. The adaptation process also creates opportunities for local stakeholders to achieve a sense of ownership and investment in the success of OR cognitive aid implementation. Third, it is important to create feedback loops [51]. The implementation pathway includes monitoring the use of the cognitive aid through debriefing after a critical event, measuring actual use, and administering provider surveys to elicit perceptions and attitudes.

### Taking advantage of “use begets use”

In contrast to surgical safety checklists which are reinforced by regular use, the rarity of OR crises make other types of exposures to the cognitive aid necessary to support familiarity and motivation to use these tools repeatedly. Cognitive aids present multiple opportunities for clinicians to use them. Through the survey, we examined multiple different uses: simulated crises, preparing for a complex case, debriefing after a crisis, or reviewing for educational purposes, as well as use during clinical crises. Having multiple types of uses within a facility was associated with more successful implementation. This suggests that use begets use.

### Sustainability

Our results indicate that 9.6% of survey respondents reported not sustaining use of the cognitive aid. While our goal was to study the implementation of OR cognitive aids, we recognize that many innovations fail in the long term, and understanding factors that sustain use is just as important [52]. Gillespie and Marshall [30] found that sustained use of surgical safety checklists for routine use was more often achieved when physicians were actively engaged and leading implementation. While research is needed to understand the factors that promote the sustainability of OR cognitive aids for crises, we believe that the use of OR cognitive aids can be sustained through multiple uses of the tools such as drills and educational reviews and opportunities for reflection through debriefing and event monitoring.

### Limitations

This study was designed to take advantage of the ability to reach thousands of potential implementers of cognitive aids and survey them in large numbers at reasonable effort and cost to learn from their implementation experience. Research based on surveys can be influenced by many biases including social desirability bias, biases in who chooses to respond perhaps favoring successful implementers, the reality that the outcome measure is a perception rather than an actual measurement, and same-source bias since dependent and independent variables came from a single respondent. Our analysis is also limited to reporting associations and not causality. Since the survey distribution was based on email addresses and we wished to protect the confidentiality of respondents, there is a small possibility of more than one respondent from a single facility. We were not able to adjust for clustering. Since the analysis is reflective of implementation experience surrounding two specific tools, the experience with other tools may be different. The survey was also given at a single point in time, limiting our ability to better understand sustainability. We believe that these biases should be considered in the interpretation of the analysis but that the learning from front-line implementers is of great value.

## Conclusion

Even though OR cognitive aids are simple tools to improve care, they are only effective when used appropriately. The tools appear to have benefits that go beyond being aids to memory. Clinicians who reported the tools were used during clinical crises also reported improved teamwork and communication. Respondents who perceived that the OR cognitive aid was used for patients in their facility also perceived that the tool positively impacted care, in multiple ways. The ultimate expression of support by clinicians of this tool, in theory, was confirmed through their indication of whether they would want it used if they were having an OR crisis. Of the 368 OR clinicians and staff, 90% would want the tool used in their own care, regardless of whether their facilities were currently successful in implementing the tool. Given the evidence base for efficacy from simulation studies and the implementation successes of multiple facilities achieving effective use during clinical crises, this statement should motivate facilities to take the necessary steps to ensure the broad and appropriate use of OR cognitive aids during crises. Our study provides some guidance derived from real-world experience that we hope will improve efforts to successfully adopt and implement these tools and others like them, to provide the best care to patients in challenging clinical environments.

## Additional files

Additional file 1: OR cognitive aid survey. (DOCX 35 kb)

Additional file 2: Composite score component variables tested for unadjusted association with using the tool regularly (more successful implementation) vs. not (less successful implementation). (DOCX 37 kb)

Additional file 3: Association of reported impact with using the tool regularly (more successful vs. less successful implementation). (DOCX 20 kb)
