# Supplementary material for: Factors associated with the use of cognitive aids in operating room crises: a cross-sectional study of US hospitals and ambulatory surgical centers
Source: Implement Sci. 2018 Mar 26;13:50. doi: 10.1186/s13012-018-0739-4 (PMC5870083; doi:10.1186/s13012-018-0739-4)
Supplement: Supplementary file 1 — OR cognitive aid survey. (DOCX 35 kb) [file 13012_2018_739_MOESM1_ESM.docx]

**Additional file 1.** OR cognitive aids survey

**SURVEY 1:** Target group: downloaded tools but did not implement

| **A. Background Information**  The following questions ask for background information on you and the facility in which you intended to use the tools you downloaded. If you intended to use the tools **in more than one facility, please pick the facility where you are furthest along in your implementation**. |
| --- |
| 1. What type of health facility are you reporting on in this survey?  - Hospital - Ambulatory Surgery Center - Other, please specify:________________________ |
| 1. What is your primary professional role at this facility?  - Anesthesiology   - Anesthesiologist Chair/Director → *go to question A2.1*   - Attending Anesthesiologist (but not Chair/Director) → *go to question A2.1*   - Anesthesia resident orfellow   - Certified registered nurse anesthetist or Anesthesiologists Assistant → *go to question A2.1*   - Other position; please specify:____________________ - Surgery   - Surgery Chair/Director → *go to question A2.1*   - Attending Surgeon (but not Chair/Director) → *go to question A2.1*   - Surgical resident or fellow   - Physician assistant   - Other position; please specify:____________________ - OR staff   - Nursing Director/Administrator → *go to question A2.1*   - Surgical nurse → *go to question A2.1*   - Surgical technologist   - Nursing student   - Other position; please specify:____________________ - Other   - Hospital administrator →*go to question A2.1*   - Quality improvement professional   - Researcher   - Medical student   - Other specialty MD *go to question A2.1*   - Other position; please specify:____________________   **A2.1**What other roles, if any, do you play in your institution? **(Please check all that apply)**   - Leaders in implementation of these tools - Administration and Quality - OR Director - Hospital senior management - Quality improvement professional - Simulation faculty - Nursing educator - Provider of patient care - Other position; please specify:____________________   **A2.2** What best describes your employment at the facility?   - Employed by facility or university - Employed by physician owned practice - Employed by corporate practice - Independent solo practice |
| 1. How many years have you worked in this role (at any facility)?  - <5 years - 6-10 years - 10-20 years - >20 years |
| 1. Approximately how many operating rooms (excluding non-OR procedural rooms) does your facility have?  - 0-4 - 5-15 - 16-30 - >30 - I don’t know |
| 1. Are there anesthesiology or surgical residents at your facility?  - Yes - No |
| 1. Has your facility implemented any of the following patient safety/QI initiatives? (**Please select all that apply**)  - WHO Safe Surgery Checklist - Simulation training - Communication and teamwork training - Protocol for handoffs - Emergency drills |
| **B. Tools for Emergencies in the Operating Room**  The following questions ask about your use of cognitive aids for emergencies in the operating room. They include tools such as Ariadne Labs’ OR Crisis Checklists, Stanford’s Perioperative Emergency Manual, the Society for Pediatric Anesthesia’s Pediatric Anesthesia Checklists, the American Society of Regional Anesthesia and Pain Medicine’s Local Anesthetic Toxicity Checklist, among others. We will refer to them as ‘tools’ in this survey. |
| 1. Which tool did you download from the website(s)? [Note: This could have a pop-up picture of the actual tools] (**Please select all that apply**)  - OR Crisis Checklists, Ariadne Labs, Harvard University - Perioperative Emergency Manuals, Stanford University - Other |
| 1. Which of the following reasons influenced your decision to download the tool for use in emergencies in the operating room? (**Please select all that apply**.)  - My department and/or institution began a (or has an ongoing) safety initiative. - I am personally working to enhance patient safety at my institution. - My institution experienced an adverse event or a “near miss.” - I am interested in learning more about patient safety measures. - Other; please describe___________________________ |
| 1. What is the stage of use of the tool in your facility? (**Please select one**.) If you intended to use the tool in more than one facility, please pick the facility where you are furthest along in your implementation.  - I downloaded the tool, but it is not in our operating rooms. - We are in the process of deploying the tool to our operating rooms. - The tool is in ORs but clinicians are not using it. - The tool is in the ORs and clinicians used it initially but we have not been successful in sustaining its use. - Clinicians use the tool in ORs when appropriate. - Our facility currently uses the tool in our operating rooms *and* expanded the use of OR cognitive aids to areas outside of our operating rooms (procedural areas, bedside, etc.). |
| **C. Barriers and facilitators**  The following questions ask about challenges you faced in working to implement the tool as well as factors that would have enabled your implementation of the tool. |
| \| 1. Which of the following factors *hindered* implementation of the tool in your facility? **(Please select only three).** \| Most important \| Second most important \| Third most important \| \| --- \| --- \| --- \| --- \| \| Lack of departmental or institutional leadership support \|  \|  \|  \| \| Clinical providers resisted using the tool \|  \|  \|  \| \| Absence of a committed implementation champion \|  \|  \|  \| \| Lack of knowledge of how best to train staff \|  \|  \|  \| \| Unavailability of time for staff training \|  \|  \|  \| \| Lack of institutional commitment to improving patient care \|  \|  \|  \| \| We were unable to easily customize the tool to suit my facility \|  \|  \|  \| \| My department/facility found the content or design of the tool unsatisfactory for our use. \|  \|  \|  \| \| Other, please describe:_________________________________________________________________________________________________________________________________________________________________________ \|  \|  \|  \| \|  \|  \|  \|  \| \| 1. Which of the following factors do you feel would be most *helpful* in implementing the tool in your institution? \| Most important \| Second most important \| Third most important \| \| The support of my department or institution’s leadership \|  \|  \|  \| \| A committed implementation champion(s) \|  \|  \|  \| \| The guidance or coaching to know how best to train staff \|  \|  \|  \| \| The time to train staff \|  \|  \|  \| \| Working in an institution committed to improving patient care \|  \|  \|  \| \| Growing evidence base about the importance of cognitive aids in the management of OR crises \|  \|  \|  \| \| Other, please describe:_____________________________________________________________________________________________________________________________________________________________________________________________________________________ \|  \|  \|  \| |
|  |
|  |
| **D. Additional information**  The following questions asks about your perceptions of the tool and follow-up information.. |
| 1. If I were having an operation that had an intraoperative emergency, I would want this tool to be used.  - Strongly disagree - Disagree - Neither agree nor disagree - Agree - Strongly agree |
| 1. Please share any further information that may help us understand your experience with implementing and/or using the tool.   _________________________________________________________________________________________________________________________________________________________________________________________________________________________________________________________________________________________________________________________________________________________________________________________________________________________________________________________________________________________________________________________________________________________________ |
| 1. Your experience is immensely valuable to us in improving our work. Can we contact you for a 30 minute interview to learn more about your experience with implementing the tool?  - Yes [ask for name and contact information and facility name - No |
| 1. What is the name of the facility in which you intended to use the tool you downloaded (optional )  - Facility name:_________________________________ |
| 1. This project is funded by the Agency for Healthcare Research and Quality (AHRQ) which requires all research to ask the following demographic questions. If you prefer not to answer either of these questions, please select “Decline to answer.”   Which category best describes your race?   - Hispanic or Latino - American Indian or Alaska Native - Asian - Black or African American - Native Hawaiian or Other Pacific Islander - White - Multiracial - Decline to answer - Unknown   What is your gender?   - Female - Male - Decline to answer |

**Thank you very much for your participation. Your response is very important to us.**

Additional resources on these tools are available on the EMIC website [link]

**SURVEY 2:** Target group: Downloaded OR Crisis Checklists/Emergency Manual and:

A) Currently implementing, or

B) Attempted implementation but failed, or

C) Successfully implemented.

This document includes the survey questions that are only asked of the target group described above. The question preceding B4 in the survey #1 document is: What is the stage of use of the tool in your facility?

| **B4.** After downloading the tools, when did you start the first steps to implement the tool?   - <3 months ago - 3-6 months ago - 6-12 months ago - >12 months ago |
| --- |
| **C. Implementation Process**  The following questions ask about activities your team may or may not have used to implement the tool. |
| **C1.** Please indicate which, if any, activities you undertook to implement the tool in your facility? (Please select all that apply.) |
| **C1.1** Has the tool been placed in at least one OR? Yes/No |
| **C1. 2** Has the tool been presented at staff, physician or departmental meetings? Yes/No |
| **C1. 3** Has your facility established a multidisciplinary team to review the tool? Yes/No  *If yes, go to C1.4.1*  **C1.4.1**Who was part of the multidisciplinary team? (Please select all that apply.)   - Anesthesiologist - Surgeon - Certified registered nurse anesthetist/Anesthesia Assistant - Other Nurse - Physician’s Assistant - Surgical tech - Administrator - QI officer - Other; please specify:_____________ |
| **C1. 4** Did you customize the tool to your facility’s local context? Yes/No  *If yes, go to C1.5.1*  **C1.5.1** How has your facility customized the tool to suit local context? (Please select all that apply.)   - Added local contact telephone numbers - Modified to match existing hospital/facility protocols - Added additional critical events to the tool - Edited the steps or changed the order of the management steps - Modified instructions for use of equipment - Changed medications/names to align with facility formulary - Other; please specify:_______________ |
| **C1. 5** Did your facility pilot test the tool (e.g. role-playing exercises to ensure that customizations worked with your clinical workflow and environment; trying it in one operating room before expanding to other operating rooms)? Yes/No |
| **C1. 6** Has your facility trained people working in the use of the tool? Yes/No  *If yes, go to C1.7.1*  **C1.6.1** Please indicate who was trained and the type of training that was provided (**Please select all that apply.)**   \|  \| Didactic presentations \| On-line tutorials \| Emergency drills in the operating room \| Emergency drills in a Simulation Center \| Other \| \| --- \| --- \| --- \| --- \| --- \| --- \| \| Anesthesia \|  \|  \|  \|  \|  \| \| Surgery \|  \|  \|  \|  \|  \| \| OR staff \|  \|  \|  \|  \|  \| |
| **C1. 7** Does your facility provide ongoing/routine training on the effective use of this tool? Yes/No  *If yes, please go to C1.8.1*  **C1.8.1** How does your facility provide ongoing/routine training of the tool? (Please select all that apply.)   - Drills - On line curriculum - Coaching - Peer feedback - Integrate into Case conference or Morbidity and Mortality conference - Other, please specify:____________ |
| **C1.8** Does your facility monitor the use of tool? Yes/No  *If yes, go to C1.8.1*  **C1.8. 1** Which of the following do you use to monitor the use of the tool? (Please select all that apply.)   - Electronic medical records - Chart review - Staff survey - Event debriefing - Other; please specify:_______________ |
| C1.9 Has your facility expanded the use of the tool to other areas in the hospital where anesthesia is being administered? Yes/No |
| **D. Barriers and facilitators**  The following questions ask about challenges you faced in working to implement the tool as well as factors that enabled your implementation of the tool. |
| \| 1. What have been the most important factors that have *enabled* your implementation of the tool at your facility? \| Most important \| Second most important \| Third most important \| \| --- \| --- \| --- \| --- \| \| The support of my department or institution’s leadership \|  \|  \|  \| \| A committed implementation champion \|  \|  \|  \| \| The guidance or coaching to know how best to train staff \|  \|  \|  \| \| The time to train staff \|  \|  \|  \| \| Working in an institution committed to improving patient care \|  \|  \|  \| \| Other, please describe:_________________________________________________________________________________________________________________________________________________________________________ \|  \|  \|  \| |
| \| 1. What have been the most important *challenges* you faced in implementing the tools? \| Most important \| Second most important \| Third most important \| \| --- \| --- \| --- \| --- \| \| Lack of departmental or institutional leadership support \|  \|  \|  \| \| Clinical providers resisted using the tool \|  \|  \|  \| \| Absence of a committed implementation champion \|  \|  \|  \| \| Lack of knowledge of how best to train staff \|  \|  \|  \| \| Unavailability of time for staff training \|  \|  \|  \| \| Lack of institutional commitment to improving patient safety programs \|  \|  \|  \| \| We were unable to easily modify the tool to suit my facility \|  \|  \|  \| \| My department/facility found the content or design of the tools unsatisfactory for our use. \|  \|  \|  \| \| Other, please describe:_________________________________________________________________________________________________________________________________________________________________________ \|  \|  \|  \| |
| **E. Checklist use**  The following questions ask about how the tool is used in your facility and your perceptions about its impact in your facility. |
| 1. At my facility, the tool is used regularly during applicable clinical events.  - Strongly disagree - Disagree - Neither agree nor disagree - Agree - Strongly agree |
| 1. How is the tool used in your facility? (Please select all that apply.)  - Emergency drills in the OR or simulation center - To aid in real time management of emergencies - To prepare for a complex case - To debrief after a critical event - Educational review - Other, please specify:___________________________________________________ |
| **F. Checklist Impact**  The following question asks about your perceptions regarding the impact of the tool in your facility |
| 1. What has been the impact of bringing the tool into your operating rooms? (Please select all that apply.)  - It has improved team performance in critical event management. - It has helped to identify lack of equipment/equipment issues to deal with particular crises. - It helped to identify systems barriers to dealing with crises. - It brought disciplines together to train as teams. - It has created a system to debrief after crises occur. - It has improved communication during a crisis. - It has improved teamwork during a crisis. - It has improved patient outcomes from critical events. - There has been no impact. - Other, please describe:____________________________________________________ |
| Next question on Survey 1 is: If I were having an operation that had an intraoperative emergency, I would want this tool to be used. |
