# Supplementary material for: Factors associated with the use of cognitive aids in operating room crises: a cross-sectional study of US hospitals and ambulatory surgical centers
Source: Implement Sci. 2018 Mar 26;13:50. doi: 10.1186/s13012-018-0739-4 (PMC5870083; doi:10.1186/s13012-018-0739-4)
Supplement: Supplementary file 2 — Composite score component variables tested for unadjusted association with using the tool regularly (more successful implementation) vs. not (less successful implementation). (DOCX 37 kb) [file 13012_2018_739_MOESM2_ESM.docx]

| **Additional file 2.** Composite score component variables tested for unadjusted association with using the tool regularly (more successful implementation) vs. not (less successful implementation) | | | | | | | | | | | | | | | | | |
| --- | --- | --- | --- | --- | --- | --- | --- | --- | --- | --- | --- | --- | --- | --- | --- | --- | --- |
|  |  | | | | | | | | |  | |  | |  |  |  |  |
|  | **Entire cohort** | | | | **Less successful implementation** | | | | **More successful implementation** | | | | | | |  | |
|  | **N=368** | | | | **N=127** | | | | **N=241** | | | | | | |  | |
| **Variables** | **n** | | **%** | | **n** | | **%** | | **n** | | | | **%** | | | **p-value** | |
|  |  | |  | |  | |  | |  | | | |  | | |  | |
| **Quality improvement initiatives score variables** |  | |  | |  | |  | |  | | | |  | | |  | |
| WHO Surgical Safety Checklist |  | |  | |  | |  | |  | | | |  | | | 0.8719 | |
| No | 95 | | 25.8% | | 32 | | 25.2% | | 63 | | | | 26.1% | | |  | |
| Yes | 263 | | 71.5% | | 91 | | 71.7% | | 172 | | | | 71.4% | | |  | |
| Missing | 10 | | 2.7% | | 4 | | 3.2% | | 6 | | | | 2.5% | | |  | |
| Simulation training |  | |  | |  | |  | |  | | | |  | | | 0.7754 | |
| No | 190 | | 51.6% | | 64 | | 50.4% | | 126 | | | | 52.3% | | |  | |
| Yes | 168 | | 45.7% | | 59 | | 46.5% | | 109 | | | | 45.2% | | |  | |
| Missing | 10 | | 2.7% | | 4 | | 3.2% | | 6 | | | | 2.5% | | |  | |
| Communication and teamwork training |  | |  | |  | |  | |  | | | |  | | | 0.0200* | |
| No | 128 | | 34.8% | | 54 | | 42.5% | | 74 | | | | 30.7% | | |  | |
| Yes | 230 | | 62.5% | | 69 | | 54.3% | | 161 | | | | 66.8% | | |  | |
| Missing | 10 | | 2.7% | | 4 | | 3.2% | | 6 | | | | 2.5% | | |  | |
| Protocols for handoffs |  | |  | |  | |  | |  | | | |  | | | 0.5208 | |
| No | 123 | | 33.4% | | 45 | | 35.4% | | 78 | | | | 32.4% | | |  | |
| Yes | 235 | | 63.9% | | 78 | | 61.4% | | 157 | | | | 65.2% | | |  | |
| Missing | 10 | | 2.7% | | 4 | | 3.2% | | 6 | | | | 2.5% | | |  | |
| Emergency drills |  | |  | |  | |  | |  | | | |  | | | <0.0001* | |
| No | 110 | | 29.9% | | 61 | | 48.0% | | 49 | | | | 20.3% | | |  | |
| Yes | 248 | | 67.4% | | 62 | | 48.8% | | 186 | | | | 77.2% | | |  | |
| Missing | 10 | | 2.7% | | 4 | | 3.2% | | 6 | | | | 2.5% | | |  | |
|  |  | |  | |  | |  | |  | | | |  | | |  | |
| Combo: WHO Surgical Safety Checklist or teamwork training or drills |  | |  | |  | |  | |  | | | |  | | | 0.0011* | |
| No | 49 | | 13.3% | | 27 | | 21.3% | | 22 | | | | 9.1% | | |  | |
| Yes | 319 | | 86.7% | | 100 | | 78.7% | | 219 | | | | 90.9% | | |  | |
|  |  | |  | |  | |  | |  | | | |  | | |  | |
| **Implementation Steps score variables** |  | |  | |  | |  | |  | | | |  | | |  | |
| Tool presented at meetings |  | |  | |  | |  | |  | | | |  | | | <0.0001* | |
| No | 58 | | 15.8% | | 36 | | 28.4% | | 22 | | | | 9.1% | | |  | |
| Yes | 309 | | 84.0% | | 91 | | 71.7% | | 218 | | | | 90.5% | | |  | |
| Missing | 1 | | 0.3% | | 0 | | 0.0% | | 1 | | | | 0.4% | | |  | |
| Multidisciplinary team |  | |  | |  | |  | |  | | | |  | | | <0.0001* | |
| No | 252 | | 68.5% | | 108 | | 85.0% | | 144 | | | | 59.8% | | |  | |
| Yes | 115 | | 31.3% | | 18 | | 14.2% | | 97 | | | | 40.3% | | |  | |
| Missing | 1 | | 0.3% | | 1 | | 0.8% | | 0 | | | | 0.0% | | |  | |
| Customize |  | |  | |  | |  | |  | | | |  | | | 0.0002* | |
| No | 229 | | 62.2% | | 95 | | 74.8% | | 134 | | | | 55.6% | | |  | |
| Yes | 137 | | 37.2% | | 31 | | 24.4% | | 106 | | | | 44.0% | | |  | |
| Missing | 2 | | 0.5% | | 1 | | 0.8% | | 1 | | | | 0.4% | | |  | |
| Pilot test |  | |  | |  | |  | |  | | | |  | | | <0.0001* | |
| No | 253 | | 68.8% | | 105 | | 82.7% | | 148 | | | | 61.4% | | |  | |
| Yes | 111 | | 30.2% | | 20 | | 15.8% | | 91 | | | | 37.8% | | |  | |
| Missing | 4 | | 1.1% | | 2 | | 1.6% | | 2 | | | | 0.8% | | |  | |
| Training |  | |  | |  | |  | |  | | | |  | | | <0.0001* | |
| No | 165 | | 44.8% | | 87 | | 68.5% | | 78 | | | | 32.4% | | |  | |
| Yes | 202 | | 54.9% | | 39 | | 30.7% | | 163 | | | | 67.6% | | |  | |
| Missing | 1 | | 0.3% | | 1 | | 0.8% | | 0 | | | | 0.0% | | |  | |
| Ongoing training |  | |  | |  | |  | |  | | | |  | | | <0.0001* | |
| No | 255 | | 69.3% | | 112 | | 88.2% | | 143 | | | | 59.3% | | |  | |
| Yes | 112 | | 30.4% | | 15 | | 11.8% | | 97 | | | | 40.3% | | |  | |
| Missing | 1 | | 0.3% | | 0 | | 0.0% | | 1 | | | | 0.4% | | |  | |
| Monitor |  | |  | |  | |  | |  | | | |  | | | <0.0001* | |
| No | 293 | | 79.6% | | 118 | | 92.9% | | 175 | | | | 72.6% | | |  | |
| Yes | 72 | | 19.6% | | 8 | | 6.3% | | 64 | | | | 26.6% | | |  | |
| Missing | 2 | | 0.5% | | 1 | | 0.8% | | 2 | | | | 0.8% | | |  | |
| Expanded use of tool |  | |  | |  | |  | |  | | | |  | | | <0.0001* | |
| No | 240 | | 65.2% | | 100 | | 78.7% | | 140 | | | | 58.1% | | |  | |
| Yes | 124 | | 33.7% | | 25 | | 19.7% | | 99 | | | | 41.1% | | |  | |
| Missing | 4 | | 1.1% | | 2 | | 1.6% | | 2 | | | | 0.8% | | |  | |
|  |  | |  | |  | |  | |  | | | |  | | |  | |
| **Ways in which tool was used score variables** |  | |  | |  | |  | |  | | | |  | | |  | |
| Emergency drills |  | |  | |  | |  | |  | | | |  | | | <0.0001* | |
| No | 192 | | 52.2% | | 85 | | 66.9% | | 107 | | | | 44.4% | | |  | |
| Yes | 172 | | 46.7% | | 38 | | 29.9% | | 134 | | | | 55.6% | | |  | |
| Missing | 4 | | 1.1% | | 4 | | 3.2% | | 0 | | | | 0.0% | | |  | |
| Prepare for complex case |  | |  | |  | |  | |  | | | |  | | | 0.0328* | |
| No | 301 | | 81.8% | | 109 | | 85.8% | | 192 | | | | 79.7% | | |  | |
| Yes | 63 | | 17.1% | | 14 | | 11.0% | | 49 | | | | 20.3% | | |  | |
| Missing | 4 | | 1.1% | | 4 | | 3.2% | | 0 | | | | 0.0% | | |  | |
| Debrief after critical event |  | |  | |  | |  | |  | | | |  | | | <0.0001* | |
| No | 291 | | 79.1% | | 115 | | 90.6% | | 176 | | | | 73.0% | | |  | |
| Yes | 73 | | 19.8% | | 8 | | 6.3% | | 65 | | | | 27.0% | | |  | |
| Missing | 4 | | 1.1% | | 4 | | 3.2% | | 0 | | | | 0.0% | | |  | |
| Educational review |  | |  | |  | |  | |  | | | |  | | | 0.7482 | |
| No | 170 | | 46.2% | | 56 | | 44.1% | | 114 | | | | 47.3% | | |  | |
| Yes | 194 | | 52.7% | | 67 | | 52.8% | | 127 | | | | 52.7% | | |  | |
| Missing | 4 | | 1.1% | | 4 | | 3.2% | | 0 | | | | 0.0% | | |  | |
|  |  | |  | |  | |  | |  | | | |  | | |  | |
|  |  |  | |  | |  | |  | | |  | | | | |  | |
|  |  |  | |  | |  | |  | | |  | | | | |  | |
|  |  |  | |  | |  | |  | | |  | | | | |  | |
