# Supplementary material for: Factors associated with the use of cognitive aids in operating room crises: a cross-sectional study of US hospitals and ambulatory surgical centers
Source: Implement Sci. 2018 Mar 26;13:50. doi: 10.1186/s13012-018-0739-4 (PMC5870083; doi:10.1186/s13012-018-0739-4)
Supplement: Supplementary file 3 — Association of reported impact with using the tool regularly (more successful vs. less successful implementation). (DOCX 20 kb) [file 13012_2018_739_MOESM3_ESM.docx]

**Additional File 3.** Association of reported impact with using the tool regularly (more successful vs. less successful implementation)

|  | **All respondents** | | **Less successful implementation** | | **More successful implementation** | |  |
| --- | --- | --- | --- | --- | --- | --- | --- |
|  | **N=368** | | **N=127** | | **N=241** | |  |
| **Variables** | **n** | **%** | **n** | **%** | **n** | **%** | **p-value** |
|  |  |  |  |  |  |  |  |
| **What has been the impact of bringing the tool into your operating room?** |  |  |  |  |  |  |  |
| 1=It has improved team performance in critical event management |  |  |  |  |  |  | **<0.0001** |
| No | 208 | 56.5% | 107 | 84.3% | 101 | 41.9% |  |
| Yes | 157 | 42.7% | 18 | 14.2% | 139 | 57.7% |  |
| Missing | 3 | 0.8% | 2 | 1.6% | 1 | 0.4% |  |
| 2=It has helped to identify lack of equipment/equipment issues to deal with particular crises |  |  |  |  |  |  | **0.0056** |
| No | 285 | 77.5% | 108 | 85.0% | 177 | 73.4% |  |
| Yes | 80 | 21.7% | 17 | 13.4% | 63 | 26.1% |  |
| Missing | 3 | 0.8% | 2 | 1.6% | 1 | 0.4% |  |
| 3=It helped to identify systems barriers to dealing with crises |  |  |  |  |  |  | 0.2009 |
| No | 290 | 78.8% | 104 | 81.9% | 186 | 77.2% |  |
| Yes | 75 | 20.4% | 21 | 16.5% | 54 | 22.4% |  |
| Missing | 3 | 0.8% | 2 | 1.6% | 1 | 0.4% |  |
| 4=It brought disciplines together to train as teams |  |  |  |  |  |  | **<0.0001** |
| No | 289 | 78.5% | 115 | 90.6% | 174 | 72.2% |  |
| Yes | 76 | 20.7% | 10 | 7.9% | 66 | 27.4% |  |
| Missing | 3 | 0.8% | 2 | 1.6% | 1 | 0.4% |  |
| 5=It has created a system to debrief after crises occur |  |  |  |  |  |  | **0.0009** |
| No | 292 | 79.4% | 112 | 88.2% | 180 | 74.7% |  |
| Yes | 73 | 19.8% | 13 | 10.2% | 60 | 24.9% |  |
| Missing | 3 | 0.8% | 2 | 1.6% | 1 | 0.4% |  |
| 6=It has improved communication during a crisis |  |  |  |  |  |  | **<0.0001** |
| No | 217 | 59.0% | 107 | 84.3% | 110 | 45.6% |  |
| Yes | 148 | 40.2% | 18 | 14.2% | 130 | 53.9% |  |
| Missing | 3 | 0.8% | 2 | 1.6% | 1 | 0.4% |  |
| 7=It has improved teamwork during a crisis |  |  |  |  |  |  | **<0.0001** |
| No | 214 | 58.2% | 105 | 82.7% | 109 | 45.2% |  |
| Yes | 151 | 41.0% | 20 | 15.8% | 131 | 54.4% |  |
| Missing | 3 | 0.8% | 2 | 1.6% | 1 | 0.4% |  |
| 8=It has improved patient outcomes from critical events |  |  |  |  |  |  | **<0.0001** |
| No | 299 | 81.3% | 116 | 91.3% | 183 | 75.9% |  |
| Yes | 66 | 17.9% | 9 | 7.1% | 57 | 23.7% |  |
| Missing | 3 | 0.8% | 2 | 1.6% | 1 | 0.4% |  |
| 9=There has been no impact |  |  |  |  |  |  | **<0.0001** |
| No | 280 | 76.1% | 57 | 44.9% | 223 | 92.5% |  |
| Yes | 85 | 23.1% | 68 | 53.5% | 17 | 7.1% |  |
| Missing | 3 | 0.8% | 2 | 1.6% | 1 | 0.4% |  |
| 10=Other |  |  |  |  |  |  | 0.0675 |
| No | 337 | 91.6% | 111 | 87.4% | 226 | 93.8% |  |
| Yes | 28 | 7.6% | 14 | 11.0% | 14 | 5.8% |  |
| Missing | 3 | 0.8% | 2 | 1.6% | 1 | 0.4% |  |
